# Heat spells and birth and peripartum pregnancy complications in Germany: a scoping review

**DOI:** 10.1007/s00404-025-08274-2

**Published:** 2026-01-09

**Authors:** Antonia Raab, Daniela Schmitz

**Affiliations:** 1 Faculty of Health, Witten/Herdecke University, Witten, NRW Germany; 2 Department for Human Medicine, Faculty of Health, Junior Professorship for Innovative and Digital Methods of Teaching and Learning in Multiprofessional Health Care, Witten/Herdecke University, Witten, NRW Germany

**Keywords:** Heat exposure, Pregnancy complications, Preterm birth, Climate change, Maternal health

## Abstract

**Purpose:**

Climate change is increasing global temperatures, with mid-latitude regions experiencing significant warming. Pregnant individuals are particularly vulnerable to heat exposure, which has been linked to adverse outcomes, such as preterm birth and low birth weight. However, research in temperate regions like Germany is scarce. This scoping review examines studies on the association between heat exposure and pregnancy complications in Germany.

**Methods:**

Following the JBI methodology for scoping reviews, a systematic literature search was conducted in eight databases, including PubMed and Scopus. Studies analyzing pregnancy complications in relation to heat events in Germany were included. Studies without systematic temperature data collection or focusing on long-term child development after birth were excluded.

**Results:**

Only four studies met the inclusion criteria. Two reported significant associations between heat exposure and preterm birth, though at different pregnancy stages. One study on low birth weight found no clear correlation, while another identified a strong link between high temperatures and pregnancy-related edema. Differences in exposure definitions and outcome measures limited comparability.

**Conclusions:**

Despite the heterogeneity in study designs and findings, the results from the small number of studies could indicate that heat exposure may contribute to adverse pregnancy outcomes in Germany, particularly preterm birth. To strengthen the evidence base, future research should focus on standardizing exposure definitions and employing robust methodologies. These findings underscore the need for public health strategies to mitigate the risks associated with rising temperatures for pregnant individuals.

## What does this study add to the clinical work?

**Table:** 

This study highlights heat exposure as a potential, yet underrecognized, risk factor for adverse pregnancy outcomes in Germany, particularly preterm birth, warranting increased clinical awareness during periods of high temperatures. For clinical practice, the findings suggest the importance of integrating heat-related risk assessment and preventive counseling into prenatal care, despite the currently limited and heterogeneous evidence base.	

## Introduction and background

As climate change progresses, it is anticipated that the global average temperature will increase [1]. This effect will be more pronounced over land than over water, with mid-latitude regions, such as Germany, experiencing an increased frequency and intensity of days of extreme heat compared to the pre-industrial era [2]. While the global mean temperature over land has increased by 1.59 °C, the rise in temperature in Germany was at 2.0 °C [3]. Individuals unable to adequately adapt to heat due to socioeconomic or physiological constraints are particularly vulnerable [2]. Pregnant individuals have only recently been recognized as part of this vulnerable group [4]. In health sciences, vulnerability refers to the susceptibility of individuals or social groups who, due to limited characteristics, capabilities, and resources, experience impairments to their well-being and face difficulties in adequately responding to and coping with external stressors [5, 6]. Vulnerability may be categorized as either general or specific; the determination of specific vulnerability is primarily contingent upon an individual’s sensitivity to stressors or conditions. The vulnerability of different demographic groups to climate events is subject to variation. Infants and children are generally considered to be vulnerable, while individuals suffering from dementia are considered depending on the severity of their impairment. People suffering from respiratory diseases are vulnerable to pollution-related exposures, and pregnant individuals and their foetuses are particularly vulnerable to heat stress.

Pregnant individuals exhibit reduced heat tolerance due to various physiological changes during pregnancy, such as an increased blood volume [7]. Pregnancy is, furthermore, associated with increased internal heat production from fetal growth. The generally augmented metabolism increases this effect. Physiological adaptation to heat through radiation from the skin via vasodilation—regardless of whether heat is internally or externally generated—is impeded due to fat deposition, the increased body mass index and the thus decreased body surface area in relation to the body mass [8]. Another factor due to which pregnant individuals are particularly vulnerable to heat stress is the possible association between increased sweating as adaptation to heat and the resulting dehydration. This poses an increased risk for early onset of labor [8].

A growing body of international research, including systematic reviews, suggests an association between heat exposure during pregnancy and adverse pregnancy outcomes, including preterm birth, low birth weight, and placental abruption [9, 10].

International studies from different regions contribute to the state of knowledge. For example, Davenport et al. investigated the impact of exposure to hot days on pregnancy outcomes in Africa, revealing that such exposure adversely affects pregnancy outcomes even after accounting for individual-level characteristics [11]. The study by Bonell et al. found that maternal heat strain particularly in pregnant agricultural workers, independent of heat stress exposure, was significantly associated with fetal strain. The authors highlight the urgent need for further research across diverse settings and populations to examine changes in placental blood flow following heat stress and their relationship with pregnancy outcomes [12]. In a Chinese study by Lin et al., the impact of ambient temperature on under-researched adverse pregnancy outcomes (APOs) such as maternal complications, high WBC, newborn hearing loss, and neonatal jaundice was examined, revealing that mean temperatures in the first trimester were positively associated with these outcomes, with effects lasting up to 10 weeks; however, the study was limited to a single city (Fuzhou), which may restrict the generalizability of the findings to other populations, highlighting the relevance of local conditions in studying climate-related impacts [13]. The study by Guo et al. demonstrates that exposure to compound and daytime-only heat waves during the week preceding delivery is associated with an increased risk of preterm birth, particularly among women living in rural areas. The authors conclude that extreme heat represents a key risk factor in the context of climate change and emphasize the need for regionally differentiated prevention strategies to protect pregnant individuals [14]. This highlights the importance of local perspectives, as urban–rural contexts and regional socioeconomic conditions substantially shape the health impacts of heat exposure.

In a population-based study, Gat et al. investigated environmental factors contributing to spontaneous preterm birth (PTB) and preterm premature rupture of membranes (PPROM), finding that the type of environmental predictor determines the nature of its association with preterm birth and that these associations are modified by ethnicity. Specifically, PPROM was more sensitive to air temperature fluctuations, particularly among Bedouin Arabs, with higher temperatures on the day before admission increasing risk (RR = 1.19 [95% CI, 1.03–1.37]), whereas Jewish women were more affected by air pollution 1–2 days before spontaneous preterm labor with intact membranes (RR = 1.025 [1.010–1.040] and RR = 1.017 [1.002–1.033]). The study concludes that high temperatures are an independent risk factor for PPROM in Bedouin Arabs, while air pollution is an independent risk factor for spontaneous PTL, highlighting that the precursors of spontaneous preterm birth differ in their sensitivity to environmental exposures [15]. Qu et al. investigated the association between extreme summer ambient heat and pregnancy complications in New York City. The study found that extreme heat events were linked to an increase in emergency department visits for pregnancy-related complications, particularly during summer and transitional months. The focus on New York City was chosen because of the availability of comprehensive health and meteorological data, which enabled precise analysis. The authors highlight the importance of incorporating local climate risks into health planning to better protect pregnant individuals [16].

In a systematic review by Pashaei Asl et al., the authors examined the impact of natural and man-made disasters on pregnancy outcomes and complications. The review included 90 studies and found significant associations between such disasters and adverse pregnancy outcomes, including preterm birth, low birth weight, small for gestational age, stillbirth, spontaneous abortion, and pregnancy-related hypertension. The authors emphasize that while it is impossible to prevent the occurrence of natural disasters and man-made disasters often occur abruptly, the negative consequences can be mitigated through enhanced prenatal care and avoidance of detrimental factors, such as smoking and alcohol consumption [17]. Veenema et al. conducted a systematic review of peer-reviewed studies examining the impact of climate change-related environmental exposures on perinatal and maternal health outcomes with focus only on the United States. Using a modified PRISMA approach, 39 studies were included from an initial pool of 768 publications. The review found that extreme heat and cold, air pollution, and natural disasters such as hurricanes, floods, and tropical storms are significantly associated with adverse maternal and neonatal outcomes. The authors conclude that climate-related environmental exposures pose substantial risks to maternal and perinatal health in the U.S. and emphasize the urgent need for further research, as well as policy and clinical actions focused on prevention, preparedness, adaptation, and education to mitigate these impacts [18]. In particular, a systematic review by Lakhani et al. found that ambient temperature and heat exposure during pregnancy in low- and middle-income countries (LMIC) are significantly associated with adverse birth outcomes—including preterm birth, low birth weight, stillbirth, and spontaneous abortion—underscoring the urgent need for longitudinal studies to confirm these associations and develop targeted interventions in LMIC contexts [19]. National studies are particularly important, as the measurement and definition of heat as well as local conditions vary considerably, and such differences in exposure assessment, outcome definition, and estimates reporting were identified as key limitations preventing meta-analyses.

Despite the numerous studies investigating the impact of extreme weather events and climatic stressors on pregnancy outcomes worldwide, data from temperate climates, such as Central Europe, and specifically Germany, remain limited, underscoring the need for further local research to generate robust evidence on risks and protective measures for pregnant individuals [20, 21].

Given the limited and heterogeneous data, this topic lends itself well to a scoping review to systematically map existing research, identify knowledge gaps, and inform future studies in the context of heat exposure and pregnancy outcomes in Germany. This lack of research is particularly concerning, as populations in temperate regions may be less physiologically adapted to extreme heat. Consequently, studies in these regions are imperative for the development of appropriate adaptation strategies and screening programs. The decision to focus on Germany is methodologically and contextually justified, as climatic conditions, healthcare systems, and population health profiles vary significantly across regions; limiting the scope to Germany allows for a more precise assessment of the existing evidence within a specific temperate climate zone. Moreover, Germany’s highly regulated healthcare system—with universal coverage and standardized prenatal care guidelines—provides a special context for analyzing heat-related pregnancy risks and drawing conclusions that are both scientifically robust and directly relevant for national public health policy and clinical practice. The overarching research question guiding this study is as follows: what associations exist between heat periods and the development of complications during pregnancy regarding the pregnant person and the fetus in Germany? A preliminary search in PubMed and Google Scholar indicated that, while a few studies could be found, to our knowledge no comprehensive reviews on this topic exist concerning Germany. The preliminary search was carried out using the following search terms: [(heat) AND (pregnancy complications)] AND (Germany). In PubMed the search yielded six results. Five of the found articles did not concern Germany or specify pregnancy complications as an outcome. In Google Scholar only one article was found that targeted Germany; however, its data concerning pregnancy complications was not generated in Germany [3, 9]. Only one study was found which researched the aim of this review; still the preliminary search found no review analyzing the scope of already existing data in Germany [20]. Thus, this study aims to conduct a scoping review to summarize existing and planned studies investigating the association between heat exposure and pregnancy complications in Germany. A scoping review was chosen over other types of reviews—such as systematic or meta-analytical reviews—because the research field on heat exposure and pregnancy outcomes in Germany is still emerging, with a limited number of heterogeneous studies and no established body of evidence suitable for quantitative synthesis. Scoping reviews are particularly appropriate for mapping the breadth and nature of available literature, examining how research has been conducted, identifying methodological gaps, and informing future, more focused reviews or primary research in this underexplored context. The findings will be synthesized to provide an overview of the current evidence and identify directions for future research.

## Methods

This scoping review was conducted following the methodology outlined by Elm et al. [22], which adheres to the JBI methodology [23]. A protocol was developed prior to the review.

### Inclusion criteria

Studies were included if they analyzed focusing birth and peripartum pregnancy complications, such as preterm birth or low birth weight, in relation to regional temperature data on heat periods. Only studies conducted within the German context and published in either German or English were considered. Studies that did not systematically assess temperature based on meteorological data or that focused on long-term child development beyond birth were excluded to maintain a clear and manageable scope aligned with the review’s primary objective: to assess the immediate impacts of heat exposure during pregnancy. Both quantitative primary studies and grey literature were included regardless of publication date to provide a comprehensive overview of the data landscape in Germany. No quality assessment of the studies included was performed. No temporal restriction was applied in this scoping review to ensure a comprehensive capture of all relevant studies, given the anticipated scarcity of research in the German context. Since the field is still emerging and no consolidated evidence base exists, excluding earlier studies could risk overlooking foundational work or historical trends in heat exposure and pregnancy outcomes.

### Sources and search strategy

An initial search was conducted on PubMed and Google Scholar to identify relevant articles from national and international sources. Keywords were extracted from titles, abstracts, and subject-specific index terms where available. Based on these terms, a comprehensive search strategy (see Appendix) was developed according to the research question. If possible, filters were applied to limit results to human studies. The search strategy was adapted for each database and translated into German to capture German-language sources as well. The following databases and information sources were searched in November 2024: CINAHL, ClinicalTrials.gov, Cochrane Library, Google Scholar (both in English and German), PubMed, Scopus, the German Clinical Trials Register, and the Karlsruhe Virtual Catalog (KVK).

Due to system limitations in the KVK, OR combinations were entered manually, and searches were filtered for German sources. In Google Scholar, search results were restricted to the first ten pages with full-text access in both language searches. The reference lists of included studies were screened for additional relevant articles. If deemed beneficial, study authors were contacted for further data.

### Study selection

All identified studies were uploaded into EndNote (EndNote 21.4), and duplicates were removed. A single reviewer conducted a title–abstract screening based on inclusion and exclusion criteria. Potentially relevant sources underwent a full-text review to determine eligibility. Reasons for exclusion were due to a global rather than Germany-specific focus, lack of relevance to pregnancy, or unavailable full text. The reference lists of the included studies were screened for additional relevant articles. The search results are presented in a flowchart following the Preferred Reporting Items for Systematic Reviews and Meta-Analyses extension for scoping reviews (PRISMA–ScR) [24].

### Data extraction

The search targeted studies involving humans that addressed exposure to hot temperatures, climate change, or extreme heat, as defined by corresponding MeSH terms in Appendix. Relevant data from the included studies were manually extracted into a table designed for this scoping review and outlined in the protocol (see Table 1). Results described in the articles but deemed irrelevant to the research question were excluded.

**Table 1:** Summary of relevant results from data extraction process (own representation)

Bibliographic reference	Context	Exposure definition	Examined complication(s)	Relevant results
Heimann, Y. et al*.*, Wir wollen nicht glauben, was wir wissen, Ärzteblatt Thüringen, 2024	Thuringia 2014–2018	Number of heat days > 30 °C out of 5 days prior to birth (1–3/5)	Relative Risk of preterm birth: Very early (< 28 WOP) early (< 32 WOP) late (< 37 WOP)	Very early: Basic risk: 0.37% 1/5: 0.41%, 2/5: 0.45%, 3/5: 0.73% Early: Basic risk: 1.10% 1/5: 0.92%, 2/5: 0.97%, 3/5: 1.61% Late: Basic risk: 7.07% 1/5: 7.65%, 2/5: 7.00%, 3/5: 7.04%
Schwalm, H., Schwangerschaftsödeme und Aussentemperatur, Klinische Wochenschrift Jg. 24/25, 1947	Offenbach on the Main (O) 1938–1943, 6345 Cases, Marburg (M), 5453 Cases	Monthly average temperature	Edema O: in pregnant individuals M: in pregnant individuals shortly before giving birth Hypertension Albuminuria	O: – Max. temperature and edema in august – Correlation coefficient (CC): 0.75 + 0.12 – CC in last 12 WOP: 0.57 ± 0.15 M: – CC: 0.79 ± 0.08 No correlation for Albuminuria + Hypertension
Wolf, J. et al*.,* The Association of Season and Temperature with Adverse Pregnancy Outcome in Two German States, a Time-Series Analysis, PLoS ONE, 2012	Brandenburg (BB), 2002–2010, 128,604 cases, Saxonia (S), 2005–2009), 162,913 cases	Average temperature for: Low birth weight: each trimester Preterm birth: First month, first trimester, last month	Low birth weight (LBW): < 2500 g, for births ≥ 37 WOP Preterm Birth (PTB): < 37 WOP Only singleton pregnancies	Little correlation with odds ratio for linear correlation: LBW (each trimester): 1. BB: 0.93, S: 0.89 2. BB: 0.91, S: 1.09 3. BB: 0.86, S: 1.15 PTB: – First month: BB: 0.94, S: 1.03 – First trimester: BB: 0.97, S: 1.06 – Last month: BB: 1.00, S: 1.00
Yüzen, D. et al*.*, Increased late preterm birth risk and altered uterine blood flow upon exposure to heat stress, *EBioMedicine,* 2023	Hamburg, 1999–2021, 42,905 cases	“Heat events” in the week before birth: max./perceived temperature in percentiles (90, 95, 98, 99) + duration (1–5 days)	Preterm Birth (Singleton, spontaneous delivery): Late: 34 + 0–36 + 6 WOP Early: 28 + 0–33 + 6 WOP Very early: 24 + 0–27 + 6 WOP	– for: 98th percentile + min. 3 consecutive days: RR 1.59 (daily max. temperature) – RR: 2.0 (perceived temperature)

The table was refined by author 1 during the data extraction process where necessary. The author column was merged with the bibliographic reference, as these are inherently linked. A newly added context column lists the regions, time periods, and number of cases examined in each study. The column previously labeled “Heat Definition” was renamed “Exposure Definition”, as some studies analyzed heat or high temperatures but did not provide a specific definition. In such cases, the recorded temperature data and classification methods were included under exposure in the table. In addition, the control group column was removed, as it was only explicitly stated in one study. In most cases, the control group consisted of cases recorded during the study period that were not exposed to the defined heat conditions. A graphical representation of events, as initially considered in the protocol, was deemed unsuitable due to the heterogeneity of study designs.

### Synthesis of results

The charted data were synthesized using a narrative approach, structured according to key outcome categories: preterm birth, low birth weight, and other pregnancy-related complications. Findings were compared across studies with attention to differences in exposure definitions, outcome measures, and methodological approaches. Due to the heterogeneity of study designs and the small number of included studies, a quantitative synthesis was not feasible. Instead, results were summarized thematically to highlight patterns, inconsistencies, and research gaps specific to the German context.

## Results

### Literature review

**Fig. 1:**
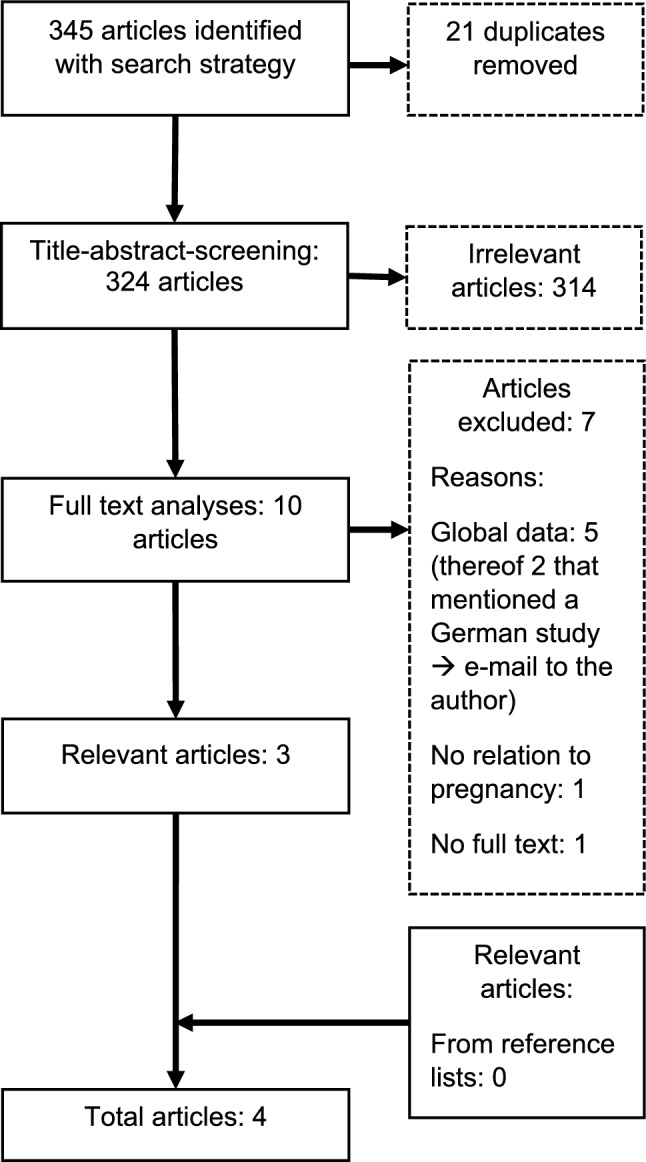
PRISMA flow diagram illustrating the identification, screening, eligibility, and inclusion process of studies examining the association between heat exposure and pregnancy complications in Germany

As part of the literature search, a total of 345 articles from seven search rounds in six databases were identified using the search strategy described in the methods section, as shown in the flowchart below (see Fig. 1). From this, 21 duplicates were removed. In the subsequent title–abstract screening, ten studies were classified as potentially relevant. These were then subjected to full-text analysis. As a result, three studies were classified as relevant [20, 21, 25].

The search of the reference lists yielded no further results. In Schwalm’s 1947 study, a detailed bibliography was missing, so additional studies referenced within the article could not be located. In two of the excluded articles, the authors referred to a Thuringian study, which had not been identified by the search strategy [26, 27]. After contacting the author, Ekkehard Schleussner, he provided the relevant study in the form of an article from the Thuringian Medical Journal, which met the inclusion criteria [28]. Thus, in total, four articles were included.

### Study characteristics

The four included studies were published in 1947 [25], 2012 [21], 2023 [20], and 2024 [27]. With the exception of the 1947 study, data from the last 25 years were analyzed. The studies originated from different regions of Germany, with two articles focusing on data from individual clinics (Marburg/Offenbach, Hamburg) and two others on data from entire federal states (Thuringia, Saxony, Brandenburg). In all articles, except for Heimann et al. [28], the number of patient cases investigated is provided, which varies significantly, ranging from 5453 in Schwalm [25] to 162,913 in Yüzen et al. [20]. The study types were quantitative studies, matching health and climate data [20], on monthly temperature and edema [25], a Logistic, time-series regression [21], and a retrospective analysis of birth data examining stillbirth and preterm birth rates [28]. In the results column, only the most relevant results for the research question are recorded. Additional findings are listed narratively below.

### Definition of exposure

To determine the temperature, all studies used data from the local meteorological service. While all studies collected daily temperature data, the analysis of these data and the timepoints at which the correlation with temperature was examined varied. Heimann et al. (2024) [28] considered heat exposures of more than 30°C for one to three consecutive days prior to birth, referred to as tropical heat days. In Schwalm et al. (1947) [25], only the temperature at the time of birth was considered, and this was extrapolated to the monthly average.

Furthermore, there was no specific focus on high temperatures, but instead looked at how any temperature, whether high or low, might relate to birth outcomes. All correlations between temperature and the endpoints were examined. In Wolf et al. (2021) [21], both high and low temperatures were retrospectively used as the basis for the investigation. Exposure was evaluated over different periods throughout pregnancy, depending on the endpoint under investigation. For low birth weight, the average temperature of each trimester was used, while for preterm birth, the average temperature of the first month, the first trimester, and the last month of pregnancy were analyzed. In Yüzen et al. (2023) [20], “heat events” lasting from 1 to 5 days were defined, similar to Heimann et al., but these events were additionally stratified by intensity using percentiles. An analysis was conducted based on the maximum daily temperatures, and another one included both temperature and humidity, which together contributed to the perceived temperature.

### Definition of endpoints

The study by Schwalm [25] differs from the other three studies in terms of the complication investigated. The researchers primarily examined the presence of edema during pregnancy and, due to its shared association with eclampsia, also included albuminuria and blood pressure values in their analysis. The more recent three studies analyzed the correlation between heat or outdoor temperature and preterm birth. In addition, Wolf et al. [21] also analyzed low birth weight as an endpoint. This study further explored the seasonality of the aforementioned endpoints. The study by Yüzen et al. [20] additionally examined a potential cause of preterm birth—a possible correlation between outdoor temperature and blood flow in the umbilical artery. These two additional endpoints in the studies by Wolf and Yüzen were not included in the present analysis, as they were not relevant to the research question.

To exclude potential confounders, only singleton pregnancies were included in the studies by Yüzen et al. [20] and Wolf et al. [21]. In addition, Yüzen et al. only considered spontaneous births and excluded cesarean sections. The other studies did not specify such procedures. Wolf et al. [21] adjusted the statistical analysis for the month, year, and, in the case of preterm births, also by the weekday and gestational age before birth. Other potential confounders, such as parity and maternal age, were only included in the statistical analysis in the study by Yüzen et al. [20].

### Edema

The study by Schwalm [25] was the only one to primarily investigate the association between outdoor temperature and edema during pregnancy. A clear correlation was found, with a Pearson correlation coefficient of + 0.75 ± 0.12. In addition, both the temperature maximum and the maximum of edema were observed in August. This relationship was further explored for the last 3 months of pregnancy, due to the increased occurrence of edema during this period, and a strong correlation of + 0.57 ± 0.15 was demonstrated. The study was then extended by incorporating a study by Henkel from 1947, which was not accessible itself but is summarized in Schwalm’s study. In Henkel’s study, edema was examined in patients who visited the clinic for childbirth, and temperature data were compared. A correlation of + 0.79 ± 0.08 was found. The investigations were subsequently supplemented by analyses of albuminuria, blood pressure, and temperature. These endpoints were selected due to the overall interest in the relationship between climatic conditions and the occurrence of eclampsia, with these three being important symptoms of the condition. However, no correlation was found between these factors.

### Low birth weight

The only study that used low birth weight as an endpoint was that of Wolf et al. [21]. In this study, little to no consistent correlation between temperature and low birth weight was found. Interestingly, the results varied between the two federal states examined—Brandenburg and Saxony—which suggests that regional factors may influence outcomes. The researchers reported odds ratios (OR) for the likelihood of low birth weight associated with a linear increase in temperature during each trimester of pregnancy. Here, little to no correlation with temperature was observed. The results also differed between the federal states of Brandenburg and Saxony. The odds ratio for a linear correlation at exposure during the first trimester was OR = 0.93 in Brandenburg and OR = 0.89 in Saxony. In the second trimester the values changed to OR = 0.91 in Brandenburg and OR = 1.09 in Saxony, and in the third trimester, the OR values were 0.86 (Brandenburg) and OR = 1.15 (Saxony). These mixed results highlight the complexity of the relationship between temperature and low birth weight and suggest that it may differ depending on regional and contextual factors.

### Preterm birth

All studies, except for Schwalm’s [25], examined a possible association between preterm birth and temperature. Among the three studies, only Wolf et al. [21] did not find a clear correlation. Here, the odds ratios for exposure in the first month of pregnancy were OR = 0.94 (Brandenburg) and OR = 1.03 (Saxony). For exposure in the first trimester, the odds ratios were OR = 0.97 (Brandenburg) and OR = 1.06 (Saxony), and in the last month of pregnancy, OR = 1.00 in both federal states.

In both Yüzen et al. [20] and Heimann et al. [28], a correlation between preterm birth and heat was found with some statistically significant correlations in the study by Yüzen et al. [20]. In Heimann et al., there was particularly an increase in the risk of preterm birth for early and very early preterm births. The baseline risk in the study was 0.37% OR, which increased to 0.41% with one heat day within 5 days before birth. With two heat days in the same period, the preterm birth risk increased to 0.73%. For early preterm births, i.e., those before the 32nd week of pregnancy, the risk increased only with exposure to three heat days within 5 days before birth, from 1.10% to 1.61%. In late preterm births, an increase was observed only with one heat day (from 7.07% to 7.65%), but not with two or three heat days.

In Yüzen et al., however, a countertrend was observed. Here, heat exposure before birth was associated with more late preterm births, especially during weeks 34–37 of pregnancy. In addition, a dose-dependent relationship was demonstrated. For instance, with heat exposure in the 98th percentile of the daily maximum temperature for at least three consecutive days, the relative risk was 1.59. When considering the perceived temperature, the relative risk increased to 2.0. Furthermore, Yüzen et al. showed a particular vulnerability of fetuses classified as female to heat stress-related preterm birth.

## Discussion

Through the conducted scoping review, four studies were identified via a literature search across eight databases, which examined a possible association between heat and pregnancy complications. When considering these four studies collectively, a strong heterogeneity is apparent, making it difficult to draw general conclusions regarding the research question. In addition, the varying definitions of exposure and endpoints further limit comparability. Heimann et al. [28] and Yüzen et al. [20], which employed similar definitions of exposure—heat periods lasting 1–5 days—each observed a significant effect on the endpoint of preterm birth, but at different times during pregnancy. In contrast, Wolf et al. [21] and Schwalm et al. [25] considered the monthly average temperature as exposure, and their research questions also deviated from those of Heimann et al. [28] and Yüzen et al. [20], making a meaningful comparison of these studies less feasible. It is possible that, due to this broad definition, the results in Wolf et al. show little correlation overall, as the potential effects of a few hot days may be averaged over the course of an entire month or trimester. A clear definition of heat as exposure could, therefore, be of great significance for future research. The standardization of heat definitions is also recommended in the systematic review by Chersich et al. [9] defined as 2 or more days with temperatures above a predefined threshold.

Overall, the findings of the studies in this review are inconsistent. When looking at international articles—especially from temperate regions—it is possible that an association between heat and certain pregnancy complications [29], particularly preterm birth, also exists in Germany. With the lack in data a final conclusion cannot be drawn at this point. On the international stage, the data are now far more diverse, showing a clearer connection between heat and preterm birth, as well as other complications [4–9]. Notably, many international clinical studies also explore associations between heat and low birth weight or stillbirths [9], with summarized results with relatively small effect sizes. Furthermore, there are studies and reviews that investigate additional potential complications [13–18]. For instance, some studies found a link between heat periods during early pregnancy and congenital heart defects [30]. The same appears to be true for hypospadias [31]. A research group from Montreal also demonstrated an increased risk of late preterm placental abruption with heat exposure [32]. For other complications, preliminary studies suggest that the impact of heat on the risk of their occurrence is unlikely. This is the case for neural tube defects and cleft palates [33, 34].

In Germany, there is a paucity of studies exploring other possible complications as endpoints. However, the data increasingly suggest that even in countries with moderate climates, such as Germany, there may be a relationship between heat and pregnancy complications [4, 9, 35–37]. A study in London, however, showed no correlation between temperature and preterm birth [38]. To make well-founded statements in Germany, systematic reviews for comparable climate regions are needed both for temperate climate zones in general and specifically for Germany. However, the primary studies required for this are currently lacking. Therefore, for future research endeavors, both the situation in temperate climates generally and more specifically in Germany would be of interest. A comprehensive analysis of births across the entire country would provide more meaningful insights than studies focusing only on specific centers. Furthermore, studies examining further pregnancy complications and their association with heat periods are also crucial to accurately assess the situation in Germany.

The studies examined also consider very few, or no, potential confounders, despite the fact that many pregnancy complications are influenced by multiple factors. This step is, therefore, of significant importance. For instance, a U.S. study found that heat exposure had a stronger impact on birth weight among Black pregnant individuals than among White pregnant individuals [39]. A systematic review also demonstrated that maternal age, BMI, smoking, and alcohol consumption affect the risk of preterm birth [40]. Furthermore, a study from Spain identified a higher risk for low and very low birth weight among pregnant individuals exposed to heat who were from a lower socioeconomic position [41]. It is evident that heat does not affect all individuals equally, and the same is true for pregnancy complications. In a study conducted in Germany, it was observed that preterm birth is influenced by the educational level and socioeconomic status of the birthing individual, and there are documented correlations between birth weight and social factors, such as the pregnant individual’s education level, family income, and living conditions [42–44]. The relationship between these risk factors in Germany and heat exposure remains to be elucidated and requires further investigation. The mechanisms underlying the potential influence of heat on pregnancy complications are not yet fully elucidated. It is noteworthy that Yüzen et al. have made a significant contribution to this research by examining changes in uterine artery blood flow due to heat exposure [20]. Further studies in this area could provide valuable insights.

## Conclusion

Should the preliminary research findings be substantiated and the hypothesis that there is a correlation between elevated ambient temperatures and pregnancy complications be validated in Germany, the question of subsequent actions will be raised. In the context of an exposure, such as heat, it is imperative to consider two fundamental pillars. The primary pillar of this framework pertains to the optimal strategies for pregnant individuals to safeguard their health during periods of elevated heat exposure. A plethora of sources have been identified that provide a comprehensive overview of potential courses of action. These include the Federal Environment Agency [45] and, with regard to pregnant individuals, the University Medical Center Hamburg-Eppendorf, as outlined in a dedicated brochure [46]. It is recommended by the aforementioned sources that measures such as adequate hydration, seeking cooler spaces, cold foot baths, or ventilating be taken. Moreover, it is recommended that screening for oligohydramnios be intensified during the heat period. However, a more profound comprehension of the underlying mechanisms would be essential to facilitate potential interventions.

The second pillar, which is of much greater importance for systematic prevention, must be the international fight against climate change as the cause of increasingly frequent heat periods at the political level [2]. Furthermore, the adaption of healthcare system structures to the anticipated changes due to the advancing climate crisis will become a task for all practitioners, whether in outpatient or clinical settings—including in the field of gynecology and obstetrics. This assertion is further validated by the recent position paper published by the German Society of Gynecology and Obstetrics, which has called upon its members to take proactive measures [47].

## Limitations

A significant limitation of this scoping review is the minimal number of eligible studies—only four met the inclusion criteria, thereby considerably diminishing the robustness and generalizability of the findings. In addition, substantial heterogeneity in the definitions of heat exposure and outcome measures across studies hindered meaningful comparison. The interpretation of results is further complicated by variations in study design, timing of exposure assessment, and regional focus further complicate the interpretation of results. The absence of a uniform methodological approach underscores the necessity for enhanced rigor and harmonization in research endeavors within this domain.

## Data Availability

No datasets were generated or analysed during the current study.

## Appendix

See Table 2.

**Table 2:** Search terms (own representation, 06.11.2024)

#1	(“Hot Temperature”[MeSH Terms] OR “Climate Change”[MeSH Terms] OR “Extreme Heat”[MeSH Terms]) AND (humans[Filter])	53,096 results
#2	((“Heat stress”[Text Word] OR “heat wave*”[Text Word] OR “Heat exposure”[Text Word] OR “Hot weather”[Text Word] OR “Hot Temperature”[Text Word] OR “temperature extreme*”[Text Word] OR “Ambient temperature”[Text Word] OR “Ambient air temperature”[Text Word] OR (“temperature”[MeSH Terms] OR “temperature”[All Fields] OR “temperatures”[All Fields] OR “temperature s”[All Fields]) OR “Climate Change”[Text Word] OR “global warm*”[Text Word] OR “Temperate climate”[Text Word]) AND “humans”[MeSH Terms] AND “humans”[MeSH Terms] AND “humans”[MeSH Terms]) AND (humans[Filter])	262,193 results
#3	(“Pregnancy Complications”[MeSH Terms] OR “Pregnancy Outcome”[MeSH Terms] OR “Maternal Health”[MeSH Terms] OR “premature birth”[MeSH Terms] OR “infant, low birth weight”[MeSH Terms] OR “Stillbirth”[MeSH Terms] OR “Abruption Placentae”[MeSH Terms]) AND (humans[Filter])	495,580 results
#4	((“gestational outcome”[Text Word] OR “gestational outcomes”[Text Word] OR “gestational outcomes”[Text Word] OR “Pregnancy outcomes”[Text Word] OR “Pregnancy complication”[Text Word] OR “Pregnancy complications”[Text Word] OR “Maternal Health”[Text Word] OR “Preterm birth”[Text Word] OR “Premature birth”[Text Word] OR “Small for gestational Age”[Text Word] OR “SGA”[Text Word] OR “Birth weight”[Text Word] OR “Preterm delivery”[Text Word] OR “Stillbirth”[Text Word] OR “Placental abruption”[Text Word] OR “Abruptio Placentae”[Text Word]) AND “humans”[MeSH Terms]) AND (humans[Filter])	326,515 results
#5	**(#1 OR #2) AND (#3 OR #4) AND German***	189 results
